# Supplementary material for: Network-wide reorganization of procedural memory during NREM sleep revealed by fMRI
Source: eLife. 2017 Sep 11;6:e24987. doi: 10.7554/eLife.24987 (PMC5593513; doi:10.7554/eLife.24987)
Supplement: Figure 1—source data 1. — The learning pattern represents all brain areas with greater activation in the MSL compared to the CTL condition during the learning practice session (S1). For each peak of activity, the anatomical label, MNI coordinates, the corrected cluster-level p value using GRF, and the associated Z-score are reported. DOI: http://dx.doi.org/10.7554/eLife.24987.004 [file elife-24987-fig1-data1.docx]

| **Anatomical label** | ***P* _cluster_** | ***Z* value** | **MNI coordinate** | | |
| --- | --- | --- | --- | --- | --- |
|  |  |  | ***X*** | ***y*** | ***Z*** |
| Left superior parietal lobule, BA7P | < 0.00001 | 6.3 | -12 | -68 | 58 |
| Right superior parietal lobule, BA7P |  | 5.1 | 16 | -66 | 56 |
| Left primary somatosensory cortex, BA2 |  | 4.8 | -40 | -36 | 50 |
| Left superior parietal lobule, BA7A |  | 5.8 | -10 | -62 | 66 |
| Left premotor cortex, BA6 | < 0.00001 | 5.3 | -32 | -2 | 52 |
| Right premotor cortex, BA6 |  | 5.2 | 30 | -4 | 50 |
| Left premotor cortex, BA6 |  | 4.9 | -36 | -6 | 58 |
| Supplementary motor area (SMA) |  | 3.7 | -4 | 4 | 48 |
| Right cerebellum, lobule VI | 0.002 | 5.0 | 28 | -56 | -28 |
| Right putamen | 0.02 | 4.5 | 26 | 12 | 2 |

**Figure 1-** **source data 1.** Summary of activation peaks related to the learning pattern. The learning pattern represents all brain areas with greater activation in the MSL compared to the CTL condition during the learning practice session (S1). For each peak of activity, the anatomical label, MNI coordinates, the corrected cluster-level p value using GRF, and the associated Z-score are reported.
