# Supplementary material for: Network-wide reorganization of procedural memory during NREM sleep revealed by fMRI
Source: eLife. 2017 Sep 11;6:e24987. doi: 10.7554/eLife.24987 (PMC5593513; doi:10.7554/eLife.24987)
Supplement: Figure 1—source data 2. — The consolidated pattern represents all brain areas with greater activation in the MSL compared to the CTL condition during the retest practice session (S2). Reporting conventions are as in Figure 1—source data 1. DOI: http://dx.doi.org/10.7554/eLife.24987.005 [file elife-24987-fig1-data2.docx]

| **Anatomical label** | ***P* _cluster_** | ***Z* value** | **MNI coordinate** | | |
| --- | --- | --- | --- | --- | --- |
|  |  |  | ***X*** | ***Y*** | ***Z*** |
| Right putamen | < 0.00001 | 5.8 | 24 | 14 | -2 |
| Right putamen |  | 4.8 | 30 | 0 | 2 |
| Left putamen |  | 4.9 | -22 | 14 | -4 |
| Left putamen |  | 4.0 | -26 | -4 | 2 |
| Left cerebellum, lobule VI/Crus I | < 0.00001 | 5.5 | -36 | -50 | -32 |
| Left cerebellum, lobule VI |  | 4.6 | -20 | -66 | -24 |
| Right cerebellum, lobule VI |  | 4.8 | 18 | -54 | -24 |
| Left superior parietal lobule, BA7P | < 0.00001 | 5.4 | -12 | -68 | 58 |
| Left premotor cortex, BA6 |  | 5.2 | -20 | -6 | 62 |
| Right premotor cortex, BA6 |  | 4.2 | 30 | -2 | 50 |

**Figure 1-** **source data 2.** Summary of activation peaks related to the consolidated pattern. The consolidated pattern represents all brain areas with greater activation in the MSL compared to the CTL condition during the retest practice session (S2). Reporting conventions are as in Figure 1- source data 1.
