# Supplementary material for: Network-wide reorganization of procedural memory during NREM sleep revealed by fMRI
Source: eLife. 2017 Sep 11;6:e24987. doi: 10.7554/eLife.24987 (PMC5593513; doi:10.7554/eLife.24987)
Supplement: Figure 3—source data 1. — Each ROI is selected based on the location of activation peaks in the learning pattern (Le), the consolidated pattern (Co), or both (Le/Co). The table shows seeds’ coordinates in MNI space (given in mm) and their anatomical labels. DOI: http://dx.doi.org/10.7554/eLife.24987.013 [file elife-24987-fig3-data1.docx]

| **Anatomical label** | **MNI coordinates** | | |  |
| --- | --- | --- | --- | --- |
|  | ***x y z*** | | | **Pattern** |
| Left superior parietal lobule, BA7P | -12 | -68 | 58 | Le/Co |
| Right superior parietal lobule, BA7P | 16 | -66 | 56 | Le |
| Left premotor cortex, BA6 | -32 | -2 | 52 | Le/Co |
| Right premotor cortex, BA6 | 30 | -4 | 50 | Le |
| Left primary somatosensory cortex, BA2 | -40 | -36 | 50 | Le |
| Supplementary motor area (SMA) | -4 | 4 | 48 | Le |
| Right putamen (rostral) | 24 | 14 | -2 | Co/Le |
| Right putamen (caudal) | 30 | 0 | 2 | Co |
| Left putamen (rostral) | -22 | 14 | -4 | Co |
| Left putamen (caudal) | -26 | -4 | 2 | Co |
| Left cerebellum, lobule VI | -20 | -66 | -24 | Co |
| Right cerebellum, lobule VI | 18 | -54 | -24 | Co/Le |

**Figure 3-** **source data 1.** Regions of interest (ROI) used in the seed-based functional connectivity analysis. Each ROI is selected based on the location of activation peaks in the learning pattern (Le), the consolidated pattern (Co), or both (Le/Co). The table shows seeds’ coordinates in MNI space (given in mm) and their anatomical labels.
