# Supplementary material for: Network-wide reorganization of procedural memory during NREM sleep revealed by fMRI
Source: eLife. 2017 Sep 11;6:e24987. doi: 10.7554/eLife.24987 (PMC5593513; doi:10.7554/eLife.24987)
Supplement: Figure 5—source data 1. — Table reports the average epoch duration and the number of epochs which were used in data analysis during the sleep scanning session in the MSL or CTL condition nights. Mean and SEM duration values are reported in minutes. p values are calculated based on paired t-statistics, and Wilcoxon signed rank test for the average duration and the number of epochs, respectively. DOI: http://dx.doi.org/10.7554/eLife.24987.020 [file elife-24987-fig5-data1.docx]

|  |  | CTL | | MSL | | MSL vs. CTL | |
| --- | --- | --- | --- | --- | --- | --- | --- |
|  |  | Mean | SEM | Mean | SEM | t | *p* |
| NREM stage 2 | Average duration | 8.40 | 1.37 | 8.28 | 1.74 | 0.05 | 0.96 |
|  | Number of epochs | 2.85 | 0.37 | 2.70 | 0.40 | - | 0.71 |
| Wake | Average duration | 10.70 | 1.82 | 11.41 | 1.70 | 0.27 | 0.79 |
|  | Number of epochs | 2.61 | 0.37 | 2.38 | 0.31 | - | 0.76 |

**Figure 5-** **source data 1.** Average duration and number of epochs used in the temporal dynamics analysis (Figure 5). Table reports the average epoch duration and the number of epochs which were used in data analysis during the sleep scanning session in the MSL or CTL condition nights. Mean and SEM duration values are reported in minutes. *p* values are calculated based on paired *t*-statistics, and Wilcoxon signed rank test for the average duration and the number of epochs, respectively.
